# A rare case of carcinoma erysipeloides/en cuirasse secondary to metastatic cutaneous apocrine adenocarcinoma

**DOI:** 10.1016/j.jdcr.2026.04.057

**Published:** 2026-05-08

**Authors:** Ruhi Kanwar, Michelle S. Lee, Ann W. Silk, Nicole R. LeBoeuf, Vinod E. Nambudiri

**Affiliations:** a Department of Dermatology, Brigham and Women's Hospital, Boston, Massachusetts; b Harvard Medical School, Boston, Massachusetts; c Center for Cutaneous Oncology, Dana-Farber Cancer Institute, Boston, Massachusetts; d Department of Medical Oncology, Dana-Farber Cancer Institute, Boston, Massachusetts

**Keywords:** carcinoma en cuirasse, carcinoma erysipeloides, cutaneous apocrine carcinoma, metastatic apocrine adenocarcinoma

## Introduction

Cutaneous apocrine adenocarcinoma is a rare cutaneous cancer with few reported cases, mostly occurring in the axilla.^1^ It is typically characterized by red-to-purple subcutaneous nodules or plaques.^2^ The diagnosis is histopathologic, requiring biopsy and immunohistochemical staining including CK7, CK20, CDX-2, thyroid transcription factor-1, CAE, MUC2, MUC5AC, estrogen receptor, and GCDFP015.^3^ Wide local excision and sentinel lymph node biopsy with adjuvant radiation therapy are often first-line in management.^2^^,^^4^ While there is no standardized treatment for metastatic disease, the literature has documented cases treated with excision, sentinel lymph node biopsy, adjuvant radiation, anti-HER2 monoclonal antibody, docetaxel, and antiestrogen therapies.

Carcinoma en cuirasse is a rare presentation of metastatic cutaneous carcinoma usually starting as erythematous papules or nodules that evolve into sclerotic plaques from lymphovascular invasion.^5^^,^^6^ It has been mostly associated with breast cancers but there also have been reports with other cancers.^5^^,^^6^ We present a rare case of a patient with progressive metastatic apocrine adenocarcinoma of the skin presenting as carcinoma erysipeloides/en cuirasse.

## Case report

A 55-year-old male with past history of metastatic cutaneous apocrine adenocarcinoma of the right axilla presented to clinic with erythematous plaques of the right chest and back.

The patient had received an initial diagnosis of adnexal carcinoma presenting as a lump in the skin of the right axillary area 6 years prior and underwent a wide local excision of a 2.1 cm primary lesion of unknown origin. He had been recommended for postoperative radiation therapy for unknown reason but had declined.

One year later, the patient developed a lump in the right axilla with right breast swelling; mammography showed an irregular mass in the right axilla and adjacent enlarged lymph node. Right axillary lymphadenectomy demonstrated metastatic carcinoma with apocrine features with lymph node involvement, extra nodal extension, and involvement of adipose tissue. Pathology was suggestive of breast origin with GATA3 positive, mammaglobin positive and GCDFP-15 positive; however, after multidisclipinary discussion of his clinical course, he was felt to more likely have primary cutaneous apocrine carcinoma. Subsequent positron emission tomography/computed tomography showed multiple fluorodeoxyglucose avid nodes in the right supraclavicular, axillary, cervical, and subpectoral area and potential sternal metastasis. The patient was started on pembrolizumab; however, his treatment course was stopped early after 2 months due to pneumonitis and subsequent progressive disease. He received radiation to the right axilla and right supraclavicular area.

**Fig 1:**
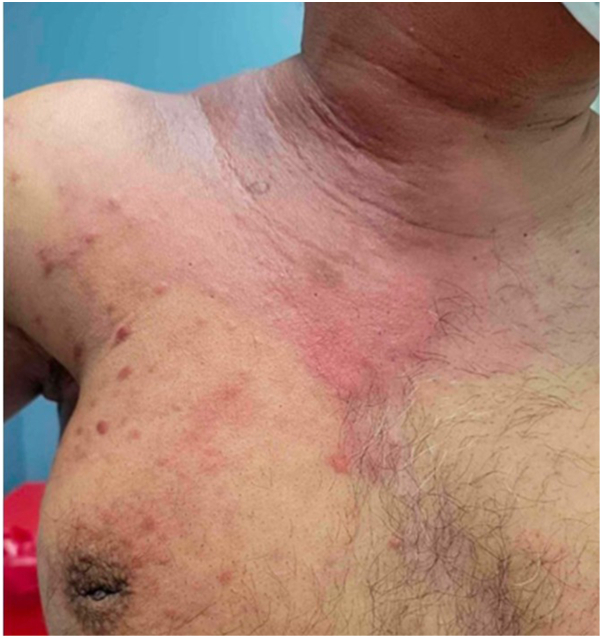
Metastatic apocrine adenocarcinoma of the skin – This figure shows the patient’s skin initial manifestations 2 years prior to diagnosis of carcinoma erysipeloides/en cuirasse. (Onset of chest and shoulder plaques)

**Fig 2:**
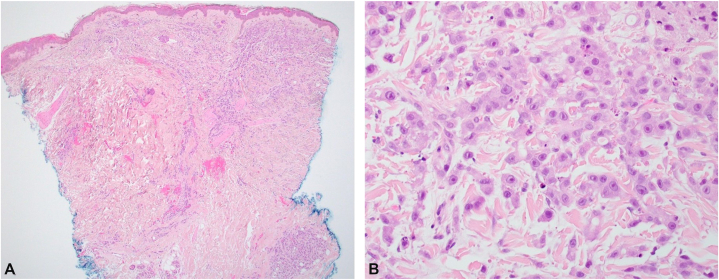
Histology slides at initial presentation of metastatic cutaneous apocrine carcinoma. **A,** Invasive carcinoma is present in the superficial to deep dermis with an infiltrative architecture. Lymphovascular invasion is present. 20× magnification. **B,** The tumor cells show apocrine features including abundant cytoplasm, central large nuclei and prominent nucleoli. 400× magnification.

Another year later, he developed indurated plaques on the right upper chest and back (Fig 1). Biopsies showed adenocarcinoma (Fig 2). Immunohistochemistry was positive for androgen receptor and HER2 (2+) with negative FISH. He was started on bicalutamide and leuprolide. After 1 year of androgen blockade therapy, PET scan showed disease progression, so subcutaneous injection of trastuzumab/hyaluronidase was added, and topical treatment with imiquimod 5% cream thrice weekly and calcipotriene 0.005% cream twice weekly for 6 weeks was also initiated for its potential to stimulate antitumor immunity.

While his PET scan showed improvement after 1.5 months, with resolution of previously seen bone metastases, and complete response at 3 and 6 months, with no fluorodeoxyglucose-avid disease, he continued to have persistent indurated, subcutaneous plaques on the right lateral chest and flank clinically consistent with malignancy. Bicalutamide and leuprolide were discontinued due to side effects per patient preference.

**Fig 3:**
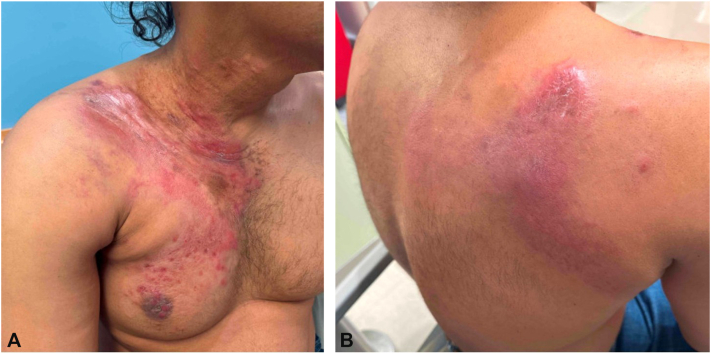
Carcinoma erysipeloides/en cuirasse – **[A, B]** This figure shows the patient’s skin manifestations at diagnosis of carcinoma erysipeloides/en cuirasse. (2 years after initial onset with more extensive indurated chest and shoulder plaques).

**Fig 4:**
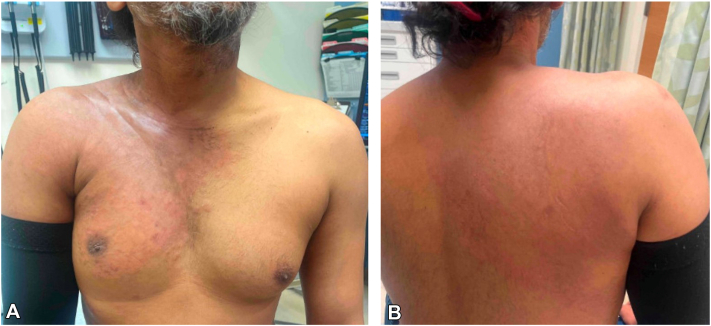
Carcinoma erysipeloides/en cuirasse – **[A, B]**, This figure shows the patient’s skin manifestations 3 months after diagnosis of carcinoma erysipeloides/en cuirasse after he was initiated on VAX014 and fam-trastuzumab deruxtecan-nxki (enhertu) treatment with significant clinical improvement.

During continued treatment with trastuzumab/hyaluronidase injections, he subsequently developed further tightness and induration of the right shoulder plaque with extension toward the neck and new scalp nodules (Fig 3). Histopathology showed metastatic aprocrine adenocarcinoma. Trastuzumab/hyaluronidase injections and topical imiquimod/calcipotriene were stopped, and he began a clinical trial of intratumoral administration of VAX014, an investigational oncolytic bacterial minicell-based therapy. An additional 1 month later, he was placed on trastuzumab deruxtecan based on the DESTINY-PanTumor02 trial for a year and 2 months with subsequent notable improvement and softening of his plaques with less skin stiffness (Fig 4).^7^ Treatment was since halted due to side effects while investigating potential alternatives, and continued monitoring of disease with PET imaging.

## Discussion

We present a rare case of progressive metastatic aprocrine adenocarcinoma of the skin with recurrence presenting as carcinoma erysipeloides/en cuirasse.

Carcinoma erisypeloides is caused by lymphatic blockage, leading to edematous erythematous plaques resembling erysipelas or cellulitis.^8^ Over time and due to progression of cutaneous metastases, the edematous plaques may evolve into the firm plaques of carcinoma en cuirasse, as seen in our patient. Carcinoma en cuirasse usually starts as erythematous papules or nodules before evolving into sclerotic plaques.^5^^,^^6^ Pathology usually shows fibrotic stroma that replace tumor cells, with remaining tumor cells observed lined up in single file within dermal collagen bundles.^5^^,^^6^

Treatment of carcinoma en cuirasse can be challenging due to decreased tissue vascularity and difficulty of chemotherapy penetration.^5^^,^^6^ There is some evidence of potential treatment with anti-HER2 humanized monoclonal antibody with taxane chemotherapy in HER2-positive metastatic cutaneous apocrine carcinoma. Trastuzumab and pertuzumab are used in combination given they bind to different epitopes of HER2, providing a more effective signal blockade. There are case reports of HER2-positive metastatic disease with induction chemotherapy with docetaxel, trastuzumab, and pertuzumab followed by chemoradiation and adjuvant trastuzumab and pertuzumab without progressive disease.^9^

First-line treatment for primary apocrine sweat gland carcinomas is wide local excision with potential adjuvant therapy. Since such tumors often express estrogen receptors and progesterone receptors, anti-estrogen therapy such as tamoxifen have been previously used and recommended.^10^ Trastuzumab-deruxtecan, an antibody drug conjugate, may be considered in the treatment of HER2 positive unresectable or metastatic disease. While treatment is challenging, this patient has shown favorable response to trastuzumab-deruxtecan, an antibody drug conjugate, which may be an option for other patients with HER2 positive unresectable or metastatic disease.

This case highlights that metastatic apocrine adenocarcinoma of the skin may present as carcinoma erysipeloides/en cuirasse, adding to our understanding of this rare cancer and the growing literature of potential etiologies for carcinoma erysipeloides/en cuirasse. The case also underscores the importance of clinical monitoring for disease, as the patient continued to have progression of skin plaques despite no evidence of disease on PET scans, and highlights the potential benefit of an antibody drug conjugate for refractory disease.

## Conflicts of interest

None disclosed.
